# Thrombophilic Variants and Clinical Outcomes in Young Ischaemic Stroke Patients Undergoing Mechanical Thrombectomy: A Retrospective Cohort Study

**DOI:** 10.3390/jcdd13100508

**Published:** 2026-10-09

**Authors:** Ebru Marzioglu Ozdemir, Gokhan Ozdemir

**Affiliations:** 1 Department of Medical Genetics, Selcuk University, Konya 42130, Turkey; 2 Stroke Center, Department of Neurology, Faculty of Medicine, Selcuk University, Konya 42130, Turkey

**Keywords:** mechanical thrombectomy, young stroke patients, hereditary thrombophilia, MTHFR C677T variant, large vessel occlusion (LVO)

## Abstract

Objective: This study aimed to characterise thrombophilic variants in young patients (≤50 years) undergoing mechanical thrombectomy (MT) for acute ischaemic stroke and to determine their clinical relevance. In addition, outcomes in young patients were compared with those in older patients to provide clinical context for age-related differences in presentation and recovery. Methods: This retrospective cohort study included consecutive patients with large-vessel occlusion stroke who achieved successful recanalisation (TICI ≥ 2b) after MT at a comprehensive stroke centre. Patients were stratified into young (≤50 years) and non-young (>50 years) groups. Clinical variables, thrombophilia markers, and functional outcomes were analysed. Results: Among 679 included patients, 76 (11.2%) were classified as young (mean age 39.2 ± 7.4 vs. 72.0 ± 11.4 years in older patients). Young patients had lower baseline NIHSS scores (14.6 ± 7.9 vs. 17.7 ± 4.7; mean difference −3.1 points) and markedly lower 1-month NIHSS scores (2.4 ± 2.3 vs. 9.5 ± 5.6; mean difference −7.1 points; both *p* < 0.001). Three-month mortality was 11.8% in young patients and 17.6% in older patients; however, this difference was not statistically significant (*p* = 0.2565). The MTHFR C677T variant was associated with lower post-procedural NIHSS scores (*p* = 0.021), while the distribution of hereditary thrombophilia markers did not differ significantly across vascular territories. Elevated homocysteine levels were associated with poorer 3-month outcomes (*p* = 0.0156). Conclusions: Young stroke patients undergoing MT demonstrated markedly better neurological and functional outcomes than older patients; however, three-month mortality did not differ significantly between the groups. Although several inherited thrombophilic variants were identified in the young cohort, most showed no consistent association with clinical outcomes.

## 1. Introduction

Ischaemic stroke in young adults commonly defined as occurring before the age of 50 represents a clinically and aetiologically heterogeneous group that requires a distinct diagnostic and therapeutic framework compared with older stroke populations [1]. While atherosclerosis and cardioembolic mechanisms predominate in elderly individuals, strokes in younger adults more frequently arise from non-atherosclerotic causes such as arterial dissection, patent foramen ovale, vasculitis, and inherited thrombophilic conditions [2]. These less typical mechanisms often complicate diagnostic evaluation and may prompt more extensive investigations.

Hereditary thrombophilic disorders including Factor V Leiden, Prothrombin G20210A, MTHFR C677T/A1298C polymorphisms, PAI-1 4G/5G, Factor XIII V34L, and hyperhomocysteinemia have been studied in relation to ischaemic stroke in young adults, particularly in cryptogenic cases where standard evaluation fails to reveal a clear aetiology [3]. The clinical significance of these markers remains uncertain, however, with inconsistent evidence regarding their associations with stroke severity, vascular distribution, recurrence risk, or functional outcomes. Existing studies often suffer from small sample sizes, heterogeneous testing strategies, and lack of uniform management [3,4]. Consequently, routine thrombophilia screening is not recommended for all stroke patients, though it may be considered in selected young individuals lacking conventional vascular risk factors or presenting with unexplained stroke mechanisms [4].

Mechanical thrombectomy has revolutionised the treatment of large-vessel occlusion (LVO) stroke, offering substantial improvements in revascularisation and functional outcomes across a wide age spectrum. Despite this, limited evidence exists regarding the relevance of inherited thrombophilic variants in young adults treated with thrombectomy. Whether such variants influence clinical presentation, procedural complications, stroke localisation, or recovery after effective reperfusion remains largely unknown, leaving a critical gap in contemporary stroke research.

To address this gap, the present study adopts a unified and coherent investigational framework. First, we examined the frequency and clinical relevance of common inherited thrombophilic variants in young adults (≤50 years) with LVO stroke who achieved successful recanalisation after mechanical thrombectomy. Second, because age is a dominant determinant of stroke prognosis, we compared young and older patients to contextualise the observed clinical outcomes within established age-related differences in stroke presentation and recovery. By analysing a uniformly reperfused cohort, this study aims to clarify both the etiological and prognostic implications of inherited thrombophilia in the modern era of endovascular stroke therapy.

## 2. Materials and Methods

This study was a retrospective, observational cohort analysis performed at an accredited comprehensive stroke centre. We examined the medical records of all consecutive patients who received mechanical thrombectomy for acute ischaemic stroke from January 2022 to April 2025.

### 2.1. Patient Selection and Classification

Individuals aged 17 years or older who exhibited acute ischaemic stroke resulting from large vascular occlusion (LVO) and underwent mechanical thrombectomy were included. To reduce variability in procedural results and to explicitly assess the impact of reperfusion on clinical prognosis and thrombophilic profiles, only patients with successful recanalisation were incorporated into the final sample. Successful recanalisation was characterised by a Thrombolysis in Cerebral Infarction (TICI) score of ≥2b, as evaluated by the interventionalist during the surgery. To mitigate potential bias, all angiographic pictures were subsequently evaluated by two independent interventionalists who were blinded to clinical results. Patients with TICI scores <2b were eliminated to prevent confounding effects associated with unsuccessful reperfusion, a recognised driver of worse outcomes. This method facilitates a more precise evaluation of age-related prognostic variables and thrombophilic influences in the context of technically effective thrombectomy. The exclusion criteria included: (1) inadequate clinical or imaging data; (2) treatment administered beyond the designated time frame; and (3) non-occlusive stroke causes (e.g., lacunar infarcts).

The study population was categorised into two age-based cohorts:

- Young stroke cohort: patients aged 50 years or younger.
- Non-young stroke cohort: individuals aged over 50 years.

The ≤50-year cutoff was selected because ‘young stroke’ constitutes a clinically distinct category in the cerebrovascular literature, and this definition aligns with prior thrombophilia stroke research; therefore, age stratification served a clinical rather than purely statistical purpose. Among successfully reperfused patients aged ≤50 years, only those with complete thrombophilia testing were retained in the final analytical cohort. Young patients with incomplete thrombophilia data were excluded, whereas thrombophilia testing was not routinely performed in the non-young cohort.

### 2.2. Data Collection

Data Acquisition Baseline demographic and clinical information were extracted from institutional stroke databases and electronic health records. The gathered variables comprised:

- Demographics: Demographic variables included age and biological sex, as recorded in the electronic medical records.
- Vascular risk factors: hypertension, diabetes mellitus, dyslipidaemia, atrial fibrillation (AF), and current smoking status.
- Stroke characteristics: site of occlusion (carotid, MCA, ACA, vertebral, basilar), presence of arterial dissection or vasculitis.
- Stroke severity: initial NIHSS (National Institutes of Health Stroke Scale) score.
- Clinical outcomes included neurological status assessed by the NIHSS at 1 month, functional outcome assessed by the modified Rankin Scale (mRS) at 3 months, and all-cause mortality at 3 months. Given the retrospective and exploratory nature of the study, no single primary endpoint was prespecified; these outcomes were analysed as complementary measures of neurological recovery, functional status, and survival.

### 2.3. Thrombophilia Subgroup

Only patients from the young stroke cohort (≤50 years) who underwent thorough thrombophilia panel testing were included in the thrombophilia analysis. Patients lacking thrombophilia tests were omitted from this study to maintain data consistency. The evaluated thrombophilic indicators comprised:

- Factor V Leiden mutation;
- Prothrombin G20210A (Factor II) mutation;
- MTHFR C677T and A1298C polymorphisms;
- PAI-1 4G/5G polymorphism;
- Factor XIII V34L polymorphism;
- Hyperhomocysteinemia.

The tests were conducted utilising polymerase chain reaction (PCR)-based genetic assays and/or biochemical analysis for homocysteine levels, following institutional standards.

In addition to demographic and clinical variables, stroke-related characteristics including the presence of arterial dissection or vasculitis and the anatomical site of arterial occlusion (MCA, ICA, ACA, basilar artery, or vertebral artery) were systematically recorded. Given the small size of the thrombophilia subgroups, these analyses were considered exploratory and hypothesis-generating rather than confirmatory. No adjustment for multiple comparisons was performed; therefore, nominal *p*-values should be interpreted cautiously.

### 2.4. Statistical Analysis

Statistical analyses were conducted using IBMS.

PSS Statistics (version 28; IBM Corp., Armonk, NY, USA). Continuous variables were reported as means ± standard deviations (SD) or medians with interquartile ranges (IQR), according to their distributional characteristics assessed using the Shapiro–Wilk test. Categorical variables were expressed as frequencies and percentages. Ordinal variables, including the modified Rankin Scale (mRS), were summarised as medians with interquartile ranges (Q1–Q3) and analysed using non-parametric methods. For the between-group comparison of 3-month mRS scores, the effect size was expressed as a rank-biserial correlation, with a 95% percentile bootstrap confidence interval based on 50,000 resamples within each age group. Positive values indicated lower mRS scores in young patients. Binary clinical outcomes were summarised using unadjusted odds ratios with 95% confidence intervals calculated on the log-odds scale. These additional calculations were performed using Python (https://www.python.org/), NumPy (https://numpy.org/), and SciPy (https://scipy.org/). 

Comparisons between the young and non-young cohorts were performed using the Mann–Whitney U test for continuous or ordinal variables, as appropriate, and either the chi-square test or Fisher’s exact test for categorical variables. In the thrombophilia subgroup, associations between individual thrombophilic markers and categorical clinical variables were evaluated using the chi-square test or Fisher’s exact test, as appropriate. For continuous or ordinal variables, the Mann–Whitney U test was used for comparisons between two independent groups, whereas the Kruskal–Wallis test was reserved for comparisons involving more than two independent groups. The prevalence of each thrombophilia marker was reported as both absolute count (*n*) and percentage (%) of the tested cohort. Given the relatively small size of the thrombophilia subgroups, these analyses were considered exploratory and hypothesis-generating rather than confirmatory. No adjustment for multiple comparisons was performed; therefore, nominal *p*-values should be interpreted cautiously. A two-sided *p*-value < 0.05 was considered nominally statistically significant in the exploratory analyses.

## 3. Results

### 3.1. Comparison Between Young and Older Stroke Patients

**Figure 1:**
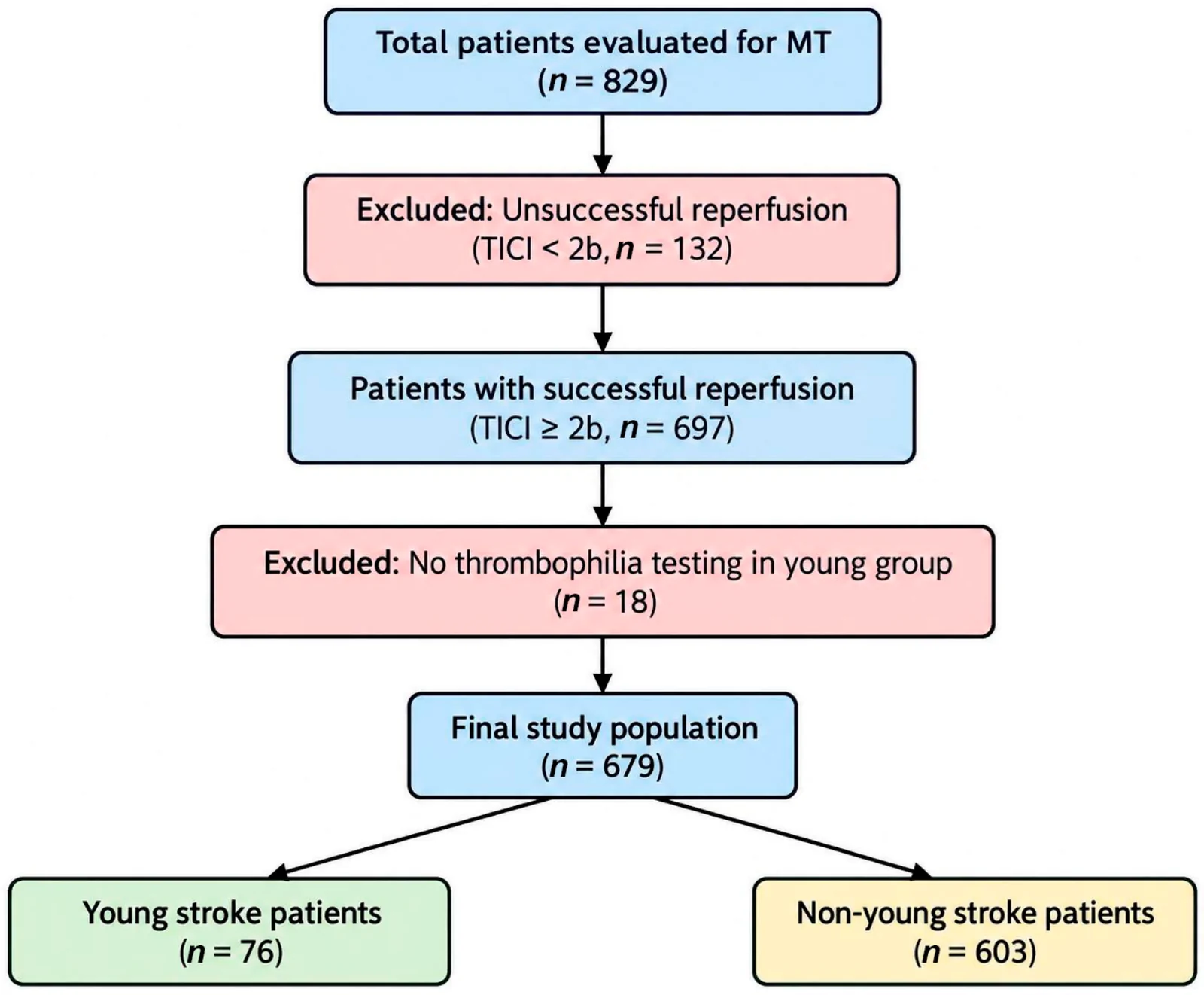
Flowchart for patient selection. A total of 829 individuals assessed for mechanical thrombectomy (MT) were screened. Patients with inadequate reperfusion, characterised by a Thrombolysis in Cerebral Infarction (TICI) score of less than 2b (*n* = 132), were eliminated. Of the 697 patients with successful reperfusion (TICI ≥ 2b), 18 individuals from the young grouping lacking thrombophilia tests were eliminated. The final study cohort consisted of 679 individuals, including 76 young stroke patients and 603 non-young stroke patients.

A total of 829 patients with acute ischaemic stroke who received mechanical thrombectomy were first evaluated. Out of these, 697 patients attained effective reperfusion (TICI ≥ 2b) and qualified for subsequent analysis. Eighteen young patients were removed due to absent thrombophilia data. The final study cohort consisted of 679 individuals, comprising 76 young stroke patients (≤50 years) and 603 non-young stroke patients (>50 years) (Figure 1).

The mean age was 39.2 ± 7.4 years in young patients and 72.0 ± 11.4 years in non-young patients. Atrial fibrillation was less frequent in young patients (10.5% vs. 27.2%, *p* = 0.0011). Diabetes mellitus was also significantly less frequent in young patients (9.2% vs. 20.4%, *p* = 0.0196), whereas the difference in dyslipidaemia did not reach statistical significance (14.5% vs. 23.9%, *p* = 0.0809). Baseline NIHSS scores were significantly lower in young patients (14.6 ± 7.9 vs. 17.7 ± 4.7, *p* < 0.001). At the one-month follow-up, young patients also had lower NIHSS scores (2.4 ± 2.3 vs. 9.5 ± 5.6, *p* < 0.001). At 3 months, median mRS scores (Q1–Q3) were 1 (1–2) in young patients and 3 (2–4) in non-young patients (*p* < 0.0001). The rank-biserial correlation was 0.560 (95% CI, 0.423–0.687), with positive values indicating lower mRS scores in young patients. Functional independence (mRS 0–2) was achieved in 61/76 young patients (80.3%) and 209/603 non-young patients (34.7%; unadjusted OR, 7.67; 95% CI, 4.25–13.82; *p* < 0.0001). Haemorrhagic transformation occurred in 8/76 young patients (10.5%) and 76/603 non-young patients (12.6%; unadjusted OR, 0.82; 95% CI, 0.38–1.76; *p* = 0.612). Three-month mortality was numerically lower in young patients, but the difference was not statistically significant (9/76 [11.8%] vs. 106/603 [17.6%]; unadjusted OR, 0.63; 95% CI, 0.30–1.30; *p* = 0.2565). Table 1 summarises the demographic characteristics, vascular risk factors, and clinical outcomes of both groups.

### 3.2. Thrombophilia Assessment in Young Stroke Patients

Only young stroke patients (≤50 years) with available thrombophilia testing were included in this subgroup analysis. The prevalence of each thrombophilic marker is summarised in Table 2.

### 3.3. Associations with Clinical Outcomes

The MTHFR C677T variant (24% prevalence) was associated with lower post-procedural NIHSS scores (median 8 vs. 12 in non-carriers), representing a 4-point reduction in neurological deficit (*p* = 0.0212). Although this may suggest a milder post-interventional course among mutation carriers, no corresponding improvement was observed in 3-month mRS scores.

Hyperhomocysteinemia (44% prevalence) demonstrated a statistically significant association with poorer 3-month functional outcomes (median mRS 3 vs. 2; *p* = 0.0156), indicating a one-point shift toward worse disability in affected patients. No thrombophilia marker including Factor V Leiden, Prothrombin G20210A, MTHFR A1298C, or Factor XIII V34L showed significant associations with dissection, vasculitis, mortality, or baseline NIHSS. Factor XIII V34L was detected in 13 patients (17.3%); no specific clinical association was identified in the exploratory analyses.

### 3.4. Associations with Vascular Territories

Significant differences in stroke severity across vascular territories were observed. Posterior circulation occlusions exhibited higher baseline NIHSS (median +4 points), higher post-procedure NIHSS, and poorer 3-month mRS outcomes compared with anterior circulation occlusions (Kruskal–Wallis *p* < 0.05). These findings were independent of thrombophilia status. Sex and hypertension also varied by occlusion site (*p* < 0.001). Male sex and absence of hypertension were more common among patients with posterior circulation strokes, whereas internal carotid artery occlusions were more prevalent in hypertensive patients and those with atrial fibrillation (*p* = 0.01). In contrast, no statistically significant differences in the distribution of Factor V Leiden, Prothrombin G20210A, MTHFR C677T, or MTHFR A1298C were observed across vascular territories.

### 3.5. Additional Clinical Characteristics

Among young stroke patients, arterial dissection was identified in 9 cases (12%), and vasculitis in 8 cases (10.6%). Occlusion sites demonstrated a heterogeneous distribution across the cohort: the middle cerebral artery (MCA) was the most frequently involved territory (38 patients, 50.7%), followed by the internal carotid artery (ICA) (17 patients, 22.7%), basilar artery (12 patients, 16%), anterior cerebral artery (ACA) (6 patients, 8%), and vertebral artery (3 patients, 4%). These distributions were incorporated into the vascular territory analyses described above to explore potential differences in stroke severity and clinical presentation across anatomical regions.

## 4. Discussion

This study examined the clinical and demographic characteristics, results, and thrombophilia profiles of young and non-young ischaemic stroke patients receiving mechanical thrombectomy. Our data indicate that younger patients demonstrated superior neurological and functional outcomes, while mortality was numerically lower but not statistically different between the age groups. Literature repeatedly reports age-related disparities in outcomes. The HERMES meta-analysis, which consolidated data from five significant randomised thrombectomy trials, found younger age as a prominent predictor of favourable 90-day functional results, irrespective of treatment mode [5]. Similarly, Roland et al. discovered that individuals of working age had superior recanalisation rates, reduced haemorrhagic sequelae, and enhanced functional independence following thrombectomy in comparison to older patients [6]. Our findings regarding neurological and functional recovery are consistent with these previous observations.

Risk factor profiles varied markedly by age group. Atrial fibrillation, diabetes mellitus, and dyslipidaemia were significantly less prevalent in the younger sample, while smoking and arterial dissection were more common, as corroborated by previous investigations like the STRATIS and SELECT registries [7,8]. Younger patients had lower baseline NIHSS scores and demonstrated better neurological status at 1 month, as well as more favourable functional outcomes assessed by the mRS at 3 months. These disparities presumably indicate different stroke mechanisms and comorbidity burdens across age groups. Arterial dissection is recognised as a primary cause of major artery occlusion in younger individuals and may provide a more advantageous haemodynamic profile, characterised by improved collateral flow and reduced microvascular damage [9]. This study’s distinctive contribution is the focused examination of inherited thrombophilia markers in young stroke patients undergoing thrombectomy. Among the thrombophilic markers evaluated, the MTHFR C677T variant was associated with lower post-procedural NIHSS scores. However, this association did not translate into a significant difference in 3-month functional outcomes. Furthermore, MTHFR C677T and the other investigated hereditary thrombophilia markers showed no significant differences in distribution across vascular territories. These findings suggest that the observed association with post-procedural neurological status should be interpreted cautiously and does not support a specific relationship between MTHFR C677T and the anatomical location of arterial occlusion. In contrast, hyperhomocysteinemia was associated with poorer functional outcomes at three months, suggesting that metabolic abnormalities related to homocysteine may have greater clinical relevance than the presence of an isolated genetic variant [10,11]. This dichotomy highlights the intricacy of interpreting isolated genetic data and underscores the necessity of considering metabolic context and timing when assessing their clinical significance. This corresponds with existing evidence connecting hyperhomocysteinemia to endothelial dysfunction, compromised collateral flow, and heightened risk of subsequent vascular events. Nonetheless, despite these correlations, extensive interventional trials targeting the reduction in homocysteine levels have not consistently shown therapeutic advantages in stroke prevention or recovery [12]. Consequently, these findings suggest a potential association between homocysteine levels and post-thrombectomy outcomes while underscoring the complexity of interpreting genetic markers such as MTHFR C677T in isolation. These isolated associations should be regarded as hypothesis-generating rather than evidence of independent prognostic effects, particularly given the small subgroup sizes, multiple unadjusted comparisons, and absence of multivariable adjustment.

Other investigated hereditary thrombophilic variants, including Factor V Leiden and Prothrombin G20210A, did not demonstrate significant associations with the evaluated clinical outcomes. In contrast, hyperhomocysteinemia was associated with poorer 3-month functional outcome, although this finding should be interpreted cautiously given the relatively small sample size and the exploratory nature of the analysis. Overall, these findings are consistent with previous studies reporting variable and often limited clinical associations between inherited thrombophilic variants and young-onset or cryptogenic ischaemic stroke [3,13,14]. Numerous prior studies have examined the correlation between thrombophilia and ischaemic stroke in young individuals, yielding contradictory findings. Pezzini et al. indicated that Factor V Leiden and Prothrombin G20210A are linked to a heightened risk of ischaemic stroke in some young patients, especially those with patent foramen ovale (PFO) or without traditional risk factors [15]. Conversely, the other investigations have identified no significant correlation between these mutations and stroke recurrence, severity, or outcome, thus challenging their routine therapeutic utility [16,17]. In young patients, the potential contribution of thrombophilic markers should also be interpreted within the broader context of competing stroke mechanisms. In particular, patent foramen ovale (PFO) and other cardioembolic sources may contribute to otherwise cryptogenic stroke and may coexist with prothrombotic states. However, the present study was not designed to evaluate interactions between PFO, specific stroke aetiologies, and thrombophilic markers; therefore, no mechanistic conclusions regarding these relationships can be drawn from our data.

A recent meta-analysis by Chiasakul et al. revealed a statistically significant correlation between inherited thrombophilias, specifically Factor V Leiden and Prothrombin G20210A variants, and an elevated risk of arterial ischaemic stroke in adults. The authors underscored that the clinical ramifications of these findings are still ambiguous and necessitate additional inquiry, especially concerning care options [3]. This is consistent with our finding that the investigated thrombophilic variants showed limited and inconsistent associations with clinical outcomes in the tested young cohort.

Overall, most of the investigated thrombophilic markers did not demonstrate consistent associations with neurological or functional outcomes. Although MTHFR C677T was associated with lower post-procedural NIHSS scores and hyperhomocysteinemia was associated with poorer 3-month functional outcomes, these isolated findings should be interpreted cautiously given the relatively small thrombophilia-tested cohort and the exploratory nature of the analyses [18].

Our data indicate that the investigated thrombophilic markers showed limited and inconsistent associations with clinical outcomes in young patients with LVO stroke undergoing mechanical thrombectomy. Multicenter prospective studies are required to elucidate the actual significance of these genetic markers in modern stroke management.

Significantly, our artery-specific subgroup analysis uncovered substantial clinical trends. Strokes in the posterior circulation correlated with increased stroke severity and poorer outcomes, corroborating previous findings that vertebrobasilar occlusions are more intricate and less amenable to reperfusion treatments. Moreover, these strokes were more prevalent in male individuals devoid of hypertension, indicating a unique non-atherosclerotic character. In contrast, ICA occlusions were significantly associated with conventional vascular risk factors, including hypertension and atrial fibrillation. Thrombophilic mutations were uniformly distributed across occlusion areas, suggesting that although these variants may affect overall stroke risk, they are improbable determinants of the vascular location of occlusion.

Our findings contribute to the ongoing discussion regarding the clinical utility of thrombophilia testing in ischaemic stroke. Although several hereditary thrombophilic variants were identified in our young cohort, they did not exert a meaningful influence on procedural success or functional outcomes after thrombectomy. However, these observations should be interpreted with caution given the relatively small size of the thrombophilia-tested subgroup (*n* = 76), which limits statistical power and precludes robust adjustment for potential confounders such as sex or hypertension. The small number of carriers of individual thrombophilic variants limited the statistical power of the subgroup analyses. Furthermore, multiple comparisons were performed without adjustment; therefore, isolated nominally significant findings should be interpreted cautiously and require confirmation in larger cohorts. Thus, the absence of clear associations should be viewed as exploratory rather than definitive. Importantly, our results are consistent with the well-established understanding that age, underlying stroke aetiology, and conventional vascular risk factors are the primary determinants of outcome in patients undergoing mechanical thrombectomy, whereas inherited thrombophilic markers appear to play a minimal and clinically inconsistent role. Taken together, these findings indicate that the investigated thrombophilic markers provided limited and inconsistent prognostic information in this cohort. Given the exploratory nature of the analyses, no conclusions regarding the clinical utility or optimal selection of patients for thrombophilia testing can be drawn from the present study. Larger multicentre studies will be necessary to validate these preliminary findings and allow adequately powered multivariable analyses to clarify any potential contributory role of thrombophilia. Additionally, the exclusion of 18 young patients because of incomplete thrombophilia testing may have introduced selection bias. Although testing was not intentionally restricted according to clinical suspicion, the thrombophilia-tested cohort may not fully represent the entire population of young patients undergoing mechanical thrombectomy. Also, effect-size estimates and corresponding 95% confidence intervals were not available for all exploratory subgroup comparisons, further limiting assessment of the magnitude and precision of the observed associations.

This study possesses multiple strengths, including a substantial thrombectomy cohort, distinct age stratification, and extensive genetic profiling. This study was not designed to assess age-adjusted effects of thrombophilia across the entire cohort. Therefore, comparisons between young and older patients should be interpreted as descriptive and contextual rather than inferential regarding thrombophilia. Nonetheless, it possesses limitations: the retrospective, single-centre design adds possible selection bias, thrombophilia testing was not consistently conducted, and long-term follow-up beyond three months was unavailable. Additional prospective investigations are required to elucidate the pathogenic and prognostic significance of inherited thrombophilia in the contemporary context of endovascular stroke therapy. Moreover, a major methodological limitation of this study is the absence of multivariable adjustment for potential confounders. The thrombophilia subgroup consisted of only 76 patients, with several individual mutations showing very low prevalence, which rendered multivariable regression models statistically unstable and prone to overfitting. As a result, the reported associations represent unadjusted relationships that may be influenced by demographic or vascular risk factors such as sex or hypertension that could simultaneously correlate with thrombophilia status and clinical outcomes. Therefore, these findings should be interpreted as exploratory, and larger multicentre cohorts will be required to establish confounder-adjusted estimates with adequate statistical power.

## 5. Conclusions

Young patients undergoing mechanical thrombectomy demonstrated more favourable neurological and functional outcomes than older patients in unadjusted comparisons. Although several inherited thrombophilic variants were identified in the young cohort, most showed no consistent association with clinical outcomes. The isolated associations observed for MTHFR C677T and hyperhomocysteinemia should be interpreted cautiously given the exploratory nature of the analyses and the relatively small thrombophilia subgroups. Larger, adequately powered prospective studies are required to clarify the clinical and prognostic relevance of thrombophilic markers in young patients undergoing mechanical thrombectomy.

## Figures and Tables

**Table 1:** Comparison of demographic characteristics, vascular risk factors, and clinical outcomes between young and non-young stroke patients undergoing mechanical thrombectomy. Data are presented as mean ± SD, median (Q1–Q3), or *n* (%), as appropriate; age ranges are provided in parentheses. Comparisons were performed using the Mann–Whitney U test for continuous or ordinal variables and the chi-square test or Fisher’s exact test for categorical variables, as appropriate.

	Young (*n* = 76)	Non-Young (*n* = 603)	*p*-Value
Age	39.19 ± 7.40 (17–50)	71.96 ± 11.42 (51–105)	—
Male sex	46/76 (60.5%)	294/603 (48.8%)	0.0531
Hypertension	36/76 (47.4%)	419/603 (69.5%)	**0.0001**
Diabetes mellitus	7/76 (9.2%)	123/603 (20.4%)	**0.0196**
Dyslipidaemia	11/76 (14.5%)	144/603 (23.9%)	0.0809
Atrial fibrillation	8/76 (10.5%)	164/603 (27.2%)	**0.0011**
Current smoker	34/76 (44.7%)	119/603 (19.7%)	**<0.0001**
Baseline NIHSS	14.57 ± 7.90	17.68 ± 4.74	**<0.0001**
NIHSS at 1 month	2.42 ± 2.26	9.5 ± 5.56	**<0.0001**
mRS at 3 months, median (Q1–Q3)	1 (1–2)	3 (2–4)	**<0.0001**
mRS 0–2 at 3 months	61/76 (80.3%)	209/603 (34.7%)	**<0.0001**
Haemorrhagic transformation	8 (10.5%)	76 (12.6%)	0.612
Mortality	9/76 (11.8%)	106/603 (17.6%)	0.2565

NIHSS—National Institutes of Health Stroke Scale; mRS—Modified Rankin Scale.

**Table 2:** Prevalence of thrombophilia markers and exploratory associations with clinical outcomes in young patients.

Thrombophilia Marker	Type	Prevalence, *n* (%)	Exploratory Clinical Association	*p*-Value
Factor V Leiden	Genetic variant	7 (9.3)	No significant association identified	—
Factor XIII V34L	Genetic variant	13 (17.3)	No significant association identified	—
MTHFR A1298C	Genetic variant	6 (8.0)	No significant association identified	—
MTHFR C677T	Genetic variant	18 (24.0)	Lower post-procedural NIHSS	0.0212
Prothrombin G20210A	Genetic variant	6 (8.0)	No significant association identified	—
Hyperhomocysteinemia	Biochemical marker	33 (44.0)	Poorer 3-month functional outcome (mRS)	0.0156

All thrombophilia subgroup analyses were exploratory and hypothesis-generating. Reported *p*-values are nominal and were not adjusted for multiple comparisons. Hyperhomocysteinemia was evaluated as a biochemical thrombophilic marker rather than a genetic variant. MTHFR-Methylenetetrahydrofolate Reductase; NIHSS—National Institutes of Health Stroke Scale; mRS—Modified Rankin Scale All subgroup analyses were exploratory and hypothesis-generating. Reported *p*-values are nominal and were not adjusted for multiple comparisons.

## Data Availability

The original contributions presented in this study are included in the article. Further inquiries can be directed to the corresponding author.
